# Business Psychology

**Published:** 1916-02

**Authors:** 


### Business Psychology.

Some time ago a chance allusion to hypnotism brought forth an eager inquiry from the patient's husband, a salesman, with reference to its availability in his business. On the other hand, another patient, also a salesman held that the furthest one kept from such influence over a prospective buyer, the better would be the results ultimately, since there would be the genuine satisfaction of actual needs and desires. Recently, we have learned that there is a well established system of psychology as applied to salesmanship and business management, with text books and formal courses in instruction, by mail and personal teaching. The object of this system is to ascertain who can and who cannot be influenced by the will of the salesman, who is best adapted to general executive functions or to carrying out details, and various other problems arising in business. From a somewhat popular lecture, we infer that this system is a mixture of phrenology, physiognomy and suggestion, and that it has a technical nomenclature of its own, derived from various sciences. Men are classified as acid and alkaline in type, though we were unable to grasp the subtle distinction, since the speaker's conception of these terms in their chemic sense was quite different from that of the chemist. They are also classified as hatchet-faced and banana-faced, meaning convex and concave profiles. Mathematic formulae are also employed to illustrate possibilities of business suasion though, as the formulae which we saw given were incorrect, we were unable to follow the speaker to his conclusions. The speaker demonstrated his points by mental vivisections of victims from the audience; one of the victims yielding complacently to the process, the other squirming and denying the analysis of his business character.

It is interesting for the medical profession to know that business men are seeking for a practical application of psychology. If, as seems to be the case, a considerable number believe that even the blind gropings of comparatively or entirely untrained men, have already secured results of practical value, it may be worth while for expert psychiatrists to look into the matter and see if something can be developed on a sound foundation of fact. If not, and the whole attempt is chimeric, that fact would save time and trouble.

